# Relevant Genes Linked to Virulence Are Required for *Salmonella* Typhimurium to Survive Intracellularly in the Social Amoeba *Dictyostelium discoideum*

**DOI:** 10.3389/fmicb.2016.01305

**Published:** 2016-08-23

**Authors:** Sebastián Riquelme, Macarena Varas, Camila Valenzuela, Paula Velozo, Nicolás Chahin, Paulina Aguilera, Andrea Sabag, Bayron Labra, Sergio A. Álvarez, Francisco P. Chávez, Carlos A. Santiviago

**Affiliations:** ^1^Laboratorio de Microbiología, Departamento de Bioquímica y Biología Molecular, Facultad de Ciencias Químicas y Farmacéuticas, Universidad de ChileSantiago, Chile; ^2^Laboratorio de Microbiología de Sistemas, Departamento de Biología, Facultad de Ciencias, Universidad de ChileSantiago, Chile

**Keywords:** *Salmonella*, *Dictyostelium*, intracellular survival, SPI-1, SPI-2, PhoPQ, T6SS, O-antigen

## Abstract

The social amoeba *Dictyostelium discoideum* has proven to be a useful model for studying relevant aspects of the host-pathogen interaction. In this work, *D. discoideum* was used as a model to study the ability of *Salmonella* Typhimurium to survive in amoebae and to evaluate the contribution of selected genes in this process. To do this, we performed infection assays using axenic cultures of *D. discoideum* co-cultured with wild-type *S.* Typhimurium and/or defined mutant strains. Our results confirmed that wild-type *S.* Typhimurium is able to survive intracellularly in *D. discoideum*. In contrast, mutants Δ*aroA* and Δ*waaL* are defective in intracellular survival in this amoeba. Next, we included in our study a group of mutants in genes directly linked to *Salmonella* virulence. Of note, mutants Δ*invA*, Δ*ssaD*, Δ*clpV*, and Δ*phoPQ* also showed an impaired ability to survive intracellularly in *D. discoideum*. This indicates that *S.* Typhimurium requires a functional biosynthetic pathway of aromatic compounds, a lipopolysaccharide containing a complete O-antigen, the type III secretion systems (T3SS) encoded in SPI-1 and SPI-2, the type VI secretion system (T6SS) encoded in SPI-6 and PhoP/PhoQ two-component system to survive in *D. discoideum*. To our knowledge, this is the first report on the requirement of O-antigen and T6SS in the survival of *Salmonella* within amoebae. In addition, mutants Δ*invA* and Δ*ssaD* were internalized in higher numbers than the wild-type strain during competitive infections, suggesting that *S.* Typhimurium requires the T3SS encoded in SPI-1 and SPI-2 to evade phagocytosis by *D. discoideum*. Altogether, these results indicate that *S.* Typhimurium exploits a common set of genes and molecular mechanisms to survive within amoeba and animal host cells. The use of *D. discoideum* as a model for host–pathogen interactions will allow us to discover the gene repertoire used by *Salmonella* to survive inside the amoeba and to study the cellular processes that are affected during infection.

## Introduction

*Salmonella* is the causative agent of foodborne gastroenteritis and is able to infect a wide range of animal hosts. The ability of *Salmonella* to cause illness is explained in part by its proficiency to survive in host cells. Relevant genes required for this process are located in pathogenicity islands such as SPI-1 and SPI-2, which encode two independent type III secretion systems (T3SS_SPI-1_ and T3SS_SPI-2_, respectively) that inject effector proteins into host cells and are critical during various stages of infection (reviewed in Haraga et al., 2008).

Although *Salmonella* is a facultative intracellular pathogen, it spends a significant part of its life cycle in the environment sharing the habitat with a variety of protozoa. These organisms feed mainly on bacteria and fungi by phagocytosis, and digestion occurs within phagolysosomes. To escape predation, some bacteria have developed sophisticated mechanisms to survive and replicate intracellularly in protozoa (Salah et al., 2009; Denoncourt et al., 2014; Hoffmann et al., 2014). In addition, the intracellular niche within protozoa protects bacteria against adverse environmental conditions, allowing these organisms to act as environmental reservoirs for proliferation and transmission of infectious bacteria to animals and humans, including *Salmonella*. Therefore, understanding the molecular mechanisms required to survive within protozoa could generate a huge impact in developing new strategies to control *Salmonella* infections.

One of the first studies on the interaction of *Salmonella* with protozoa demonstrated that *S.* Typhimurium is able to survive intracellularly in *Acanthamoeba polyphaga*, residing and multiplying within contractile vesicles (Gaze et al., 2003). This observation suggested that protozoa play an important role in the ecology of *Salmonella* in soil and aquatic environments. Later on, a number of studies established that different serovars of *Salmonella* interact with and survive within a variety of protozoa species, including *Acanthamoeba, Tetramitus, Naegleria, Hartmannella*, and *Tetrahymena* (Tezcan-Merdol et al., 2004; Wildschutte et al., 2004; Brandl et al., 2005; Wildschutte and Lawrence, 2007; Bleasdale et al., 2009; Feng et al., 2009; Rehfuss et al., 2011).

Surprisingly, only a few of these studies addressed the molecular mechanisms involved in the interaction of *Salmonella* with protozoa. In one of such studies, the authors evaluated the requirement of the *Salmonella* virulence plasmid (SVP), *spv* and *hilA* (encoding a transcriptional activator of genes in SPI-1) on the interaction of *S.* Dublin with *Acanthamoeba rhysodes* (Tezcan-Merdol et al., 2004). The authors concluded that *hilA* and SVP contribute to the interaction of *Salmonella* with *A. rhysodes*, although *hilA* (and consequently SPI-1) is expendable for bacterial internalization and survival in this model (Tezcan-Merdol et al., 2004). A second study evaluated the role played by SPI-1, SPI-2, and the PhoP/PhoQ two-component system (that regulates the expression of many virulence genes in *Salmonella*, including those in SPI-2) in the intracellular survival of *S.* Typhimurium in *A. polyphaga* (Bleasdale et al., 2009). The results of this study demonstrated that PhoP/PhoQ and SPI-2 are essential for the survival of *S.* Typhimurium in this amoeba. In contrast, SPI-1 was shown to be dispensable for this process (Bleasdale et al., 2009).

The use of social amoeba *Dictyostelium discoideum* as a model host to study the interaction with bacterial pathogens is well documented. This organism is easy to grow and maintain in the laboratory and is amenable to cell biology, genetics and biochemistry techniques. As a professional phagocyte, it can be infected with different bacterial pathogens, and relevant virulence factors in mammals have been shown to be important in the interaction with this amoeba. In addition, the existence of on-line resources like dictyBase^1^ allows easy access to genomic data and biological information such as mutant phenotypes and corresponding reference material (Kreppel et al., 2004; Basu et al., 2013; Fey et al., 2013). These advantages make *D. discoideum* a very useful model for host–pathogen interaction studies (reviewed in Bozzaro and Eichinger, 2011; Steinert, 2011; Muller-Taubenberger et al., 2013; Verma and Srikanth, 2015).

An early report suggested that *S.* Typhimurium is degraded by *D. discoideum* and is unable to survive after 2 days of infection (Skriwan et al., 2002). Then, it was reported that knockout mutants of *D. discoideum* in genes associated to autophagy support the establishment of a replicative niche for *S.* Typhimurium, suggesting that autophagy is required for degradation of this pathogen (Jia et al., 2009). Another study demonstrated that *S.* Typhimurium is pathogenic for *Dictyostelium* cells and subverts the starvation response (Sillo et al., 2011). The degree of *Salmonella* virulence to *Dictyostelium* was reported to depend on bacterial growth conditions identified in the study (Sillo et al., 2011). More recently, it was reported that *S.* Typhimurium can survive in *D. discoideum* for at least 6 h, and that chitinase activity is dispensable for this process (Frederiksen and Leisner, 2015). Therefore, *Dictyostelium* is now considered as a suitable model to study the interaction between *Salmonella* and protozoa (Verma and Srikanth, 2015).

The aim of this study was to confirm the ability of *S.* Typhimurium to survive in *D. discoideum* and to develop infection assays suitable for assessing the contribution of selected genes in this process, especially those associated with virulence in other infection models. Our results confirmed that *S.* Typhimurium is able to survive intracellularly in *D. discoideum* and indicate that this process requires functional biosynthesis of aromatic compounds, lipopolysaccharide (LPS) including a complete O-antigen, and virulence determinants SPI-1, SPI-2, SPI-6 and PhoP/PhoQ, suggesting that *Salmonella* exploits a set of conserved molecular mechanisms to survive within protozoa and animal host cells.

## Materials and Methods

### Bacterial Strains and Culture Conditions

Bacterial strains used in this study are listed in **Table 1**. All *S.* Typhimurium strains are derivatives of the wild-type, virulent strain 14028s (Fields et al., 1986). Bacteria were routinely grown in Luria-Bertani (LB) medium (10 g/L tryptone, 5 g/L NaCl, 5 g/L yeast extract) at 37°C with agitation. When required, LB medium was supplemented with ampicillin (Amp, 100 mg/L), chloramphenicol (Cam, 20 mg/L), kanamycin (Kan, 75 mg/L), or trimethoprim (Tmp, 50 mg/L). Media were solidified by the addition of agar (15 g/L).

**Table 1:** Bacterial and *Dictyostelium* strains used in this study.

Strains	Features	Source
***Salmonella* Typhimurium**		
14028s	Wild-type, virulent strain	Laboratory collection
Δ*aroA*	14028s Δ*aroA*::Tmp	Laboratory collection
Δ*phoN*	14028s Δ*phoN*::Cam	Laboratory collection
Δ*phoPQ*	14028s Δ*phoPQ*::Kan	This study
Δ*invA*	14028s Δ*invA*::Kan	Laboratory collection
Δ*ssaD*	14028s Δ*ssaD*::Kan	Laboratory collection
Δ*clpV*	14028s Δ*clpV*::Kan	Laboratory collection
Δ*waaL*	14028s Δ*waaL*::Kan	Laboratory collection
***Escherichia coli***		
B/r DBS0348878	Wild-type strain	dictyBase
***Klebsiella aerogenes***		
DBS0305928		dictyBase
***Dictyostelium discoideum***		
AX4 DBS0302402	*axeA1 axeB1 axeC1*	dictyBase
AX2 cnxA-GFP DBS0236184	*axeA2 axeB2 axeC2* pDEXRH::*act15*/*cnxA*-RSSSKLK-*gfp*(S65T)	dictyBase

### *Dictyostelium* Strains and Culture Conditions

*D. discoideum* strains AX4 (DBS0302402) and AX2 cnxA-GFP (DBS0236184) were obtained from Dicty Stock Center (Kreppel et al., 2004; Basu et al., 2013; Fey et al., 2013), and cultured according to standard protocols (Fey et al., 2007). Briefly, *D. discoideum* strains were maintained at 22°C in SM medium (10 g/L glucose, 10 g/L peptone, 1 g/L yeast extract, 1 g/L MgSO_4_ × 7H_2_O, 1.9 g/L KH_2_PO_4_, 0.6 g/L K_2_HPO_4_, 20 g/L agar), growing on a confluent lawn of *Klebsiella aerogenes* (DBS0305928). Before infection assays, amoebae were grown at 22°C with agitation (180 rpm) in liquid HL5 medium (14 g/L tryptone, 7 g/L yeast extract, 0,35 g/L Na_2_HPO_4_, 1,2 g/L KH_2_PO_4_, 14 g/L glucose, pH 6,3) in the absence of bacteria (axenic cultures). When required, media were supplemented with streptomycin (300 mg/L), ampicillin (100 mg/L) or G418 (geneticin, 10 mg/L). Prior to infection, amoebae were harvested in early exponential phase (1–2 × 10^6^ cells/mL) and centrifuged at 500 × *g* for 10 min at 4°C. The supernatant was discarded and the pellet was washed three times using Soerensen buffer (2 g/L KH_2_PO_4_, 0.36 g/L Na_2_HPO_4_ × 2H_2_O, pH 6.0). Trypan blue exclusion and counting in a Neubauer chamber was used to determine the population of viable cells.

### Standard DNA Techniques

Plasmid DNA was obtained from overnight cultures using the “QIAprep Spin Miniprep Kit” (QIAGEN), according to the manufacturer’s instructions. When required, PCR products were purified using the “QIAquick PCR Purification Kit” (QIAGEN) as recommended by the manufacturer. DNA samples were routinely analyzed by electrophoresis in 1% agarose gels (prepared in TAE buffer) and visualized under UV light after GelRed (Biotium Inc.) staining.

### Construction of Mutant Strains

*S.* Typhimurium mutants were constructed using the Lambda Red recombination method (Datsenko and Wanner, 2000) with modifications (Santiviago et al., 2009), using plasmid pCLF1 (Tmp^R^, GenBank accession number HM047090), pCLF2 (Cam^R^, GenBank accession number HM047089) or pCLF4 (Kan^R^, GenBank accession number EU629214) as template. Primers for PCR amplification were designed according to the genomic sequence of *S.* Typhimurium strain 14028s (Jarvik et al., 2010), and are listed in Supplementary Table S1. Correct allelic replacement in each mutant was confirmed by PCR using a primer flanking the 5′ end of the corresponding ORF (primers Out5 in Supplementary Table S1) and a second primer internal to the resistance cassette (pCLF4_P1_Bam or K1 in Supplementary Table S1). Finally, each mutant allele was transduced to the wild-type background using phage P22 HT105/1 int-201 (Maloy, 1990).

### Individual Infection Assay

*D. discoideum* AX4 grown axenically (∼2 × 10^7^ cells) was co-incubated with *S.* Typhimurium 14028s or *E. coli* B/r at 22°C with agitation (180 rpm) in 10 mL of Soerensen buffer using a multiplicity of infection (MOI) of 100 bacteria/amoeba. After 1 h of co-incubation, amoebae were washed three times with Soerensen buffer to remove extracellular bacteria. Then, infected cells were suspended in 10 mL of Soerensen buffer (*t* = 0) and further incubated at 22°C with agitation. Aliquots were obtained at 0, 0.5, 1, 3, 6, and 24 h post infection. Viable amoebae were determined at each time point by Trypan blue exclusion and counting on a Neubauer chamber. In parallel, infected amoebae recovered at each time point were lysed with 0.2% Triton X-100 and titers of intracellular bacteria were determined by serial dilutions and plating on LB agar. Statistical significance was determined using a two-way ANOVA with Fisher’s LSD post-test.

### Competitive Infection Assay

*D. discoideum* AX4 grown axenically (∼2 × 10^7^ cells) was co-incubated with a 1:1 mixture of *S.* Typhimurium 14028s and a defined mutant at 22°C with agitation (180 rpm) in 10 mL of Soerensen buffer using a MOI of 100 bacteria/amoeba. Extracellular bacteria were removed after 1 h of co-incubation by three sequential washes using Soerensen buffer. Then, infected cells were suspended in 10 mL of Soerensen buffer (*t* = 0) and further incubated at 22°C with agitation. Aliquots were obtained at 0, 1, 3, and 6 h post infection. Viable amoebae were determined at each time point by Trypan blue exclusion and counting on a Neubauer chamber. In addition, infected amoebae recovered at each time point were washed once in Soerensen buffer supplemented with gentamicin (10 mg/L), then washed in Soerensen buffer to remove the antibiotic, and finally lysed with 0.2% Triton X-100. Titers of intracellular bacteria were determined by serial dilutions and plating on LB agar supplemented with the appropriate antibiotics. Competitive index (CI) values were calculated as a mean ratio of mutant to wild type, normalized to the corresponding ratio in the inoculum (internalization) or at *t* = 0 (intracellular survival), and converted logarithmically. Statistical significance was determined using a two-tailed Student’s *t-*test.

### Laser Scanning Confocal Microscopy

*S.* Typhimurium 14028s and *E. coli* B/r were transformed by electroporation with plasmid pFCcGi (Addgene plasmid number 59324), encoding the red fluorescent protein mCherry expressed constitutively (Figueira et al., 2013). Axenic *D. discoideum* AX2 cnxA-GFP (∼2 × 10^7^ cells) was co-incubated with each bacteria at 22°C for 24 h in 10 mL of Soerensen buffer, using a MOI of 10 bacteria/amoeba. Images of infected cells were acquired every hour using a Zeiss LSM 710 laser scanning confocal microscope equipped with a 63x 1.4 NA optic setup. Prior to observation, cells were mounted on a thin layer of 1% agarose in PBS buffer deposited on a glass slide. To visualize GFP-associated fluorescence (amoebae), the sample was excited at 488 nm with an argon laser and emission was detected using a filter in the 493–549 nm range. To visualize mCherry-associated fluorescence (bacteria), the sample was excited at 543 nm with a HeNe laser and emission was detected using a filter in the 548–679 nm range. Images were acquired using the ZEN 2012 Black software (Zeiss), and analyzed using Fiji and ImageJ softwares (Schindelin et al., 2012; Schneider et al., 2012).

## Results

### Intracellular Survival of *S.* Typhimurium in *D. discoideum*

To determine if *S.* Typhimurium 14028s is able to survive intracellularly in *D. discoideum*, we co-incubated both organisms, and let amoebae feed on bacteria. At different time points, intracellular bacteria recovered from infected amoebae were titrated. As a control, the same procedure was performed using *E. coli* B/r, a strain commonly used as food source for this amoeba (Fey et al., 2007).

**FIGURE 1:**
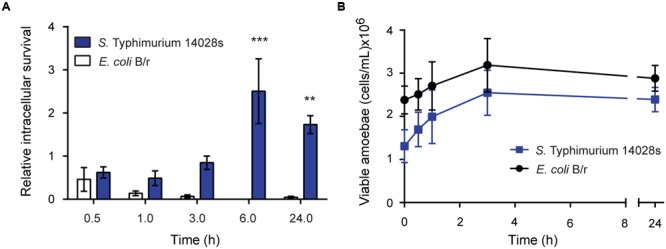
**Intracellular survival of *S.* Typhimurium 14028s and *E. coli* B/r in *D. discoideum.*** Individual infection assays were used to determine the intracellular survival of *S.* Typhimurium 14028s or *E. coli* B/r in *D. discoideum* AX4. **(A)** Intracellular survival calculated at each time point as CFU_t=x_/CFU_t=0_. **(B)** Population of viable amoebae at each time point expressed as cells/mL. Graphics shows the mean of at least three independent assays ± SEM. Statistical significance was determined using a two-way ANOVA with Fisher’s LSD post-test (^∗∗^*P* < 0.005, ^∗∗∗^*P* < 0.001).

We observed that titers of *S.* Typhimurium 14028s associated with *D. discoideum* cells remained high and constant during the first 3 h of infection. After 6 h of infection, the titer of associated bacteria reached a peak, and remained at similar level at 24 h post infection (**Figure 1A**). In contrast, *E. coli* B/r titers declined sharply after 1 h of infection, being almost under the limit of detection at 6 and 24 h of infection (**Figure 1A**). The differences observed in both cases cannot be attributed to a differential effect of infecting bacteria on cell viability since viable counts of amoeba infected with *S.* Typhimurium 14028s and *E. coli* B/r were equivalent during the course of the experiment. In both cases, the population of infected amoeba started raising at 30 min of infection, reaching a maximum level at 3 h of infection (**Figure 1B**).

To support our observations, we followed the intracellular fate of *S.* Typhimurium 14028s and *E. coli* B/r in *D. discoideum* by laser scanning confocal microscopy. To do this, bacteria constitutively expressing the red fluorescent protein mCherry from plasmid pFCcGi (Figueira et al., 2013) were co-incubated for 24 h with *D. discoideum* expressing a calnexin-GFP protein fusion (Muller-Taubenberger et al., 2001) and the interaction of bacteria and amoebae was monitored every 1 h.

**FIGURE 2:**
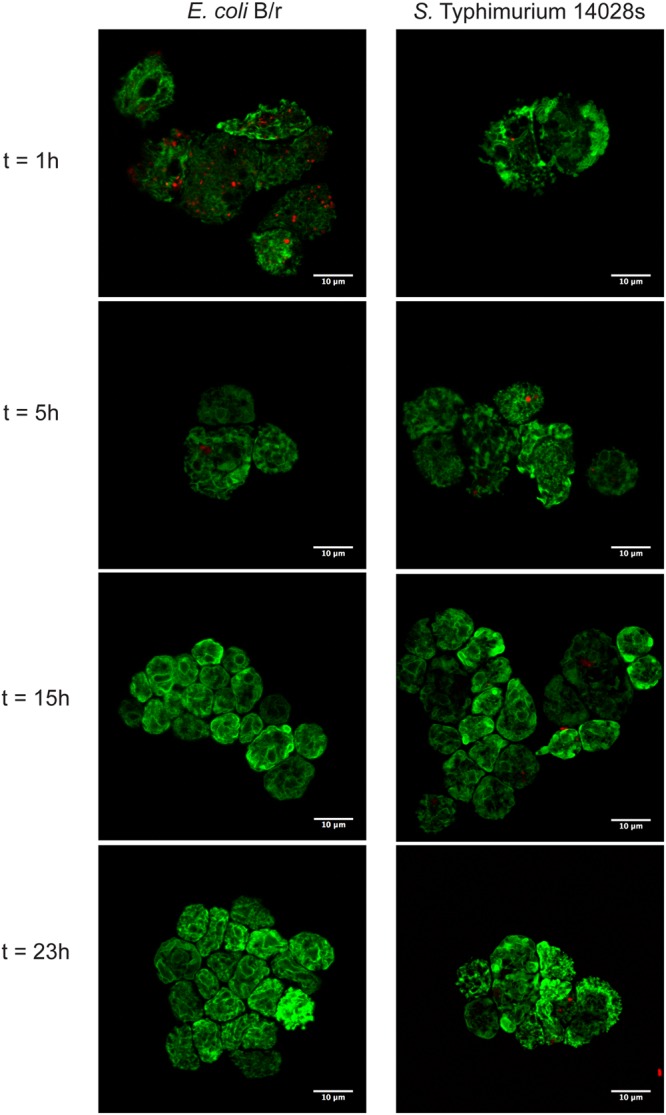
**Analysis of *D. discoideum* infected with *S.* Typhimurium 14028s and *E. coli* B/r by confocal microscopy.** Individual infection assays were used to determine the intracellular fate of *S.* Typhimurium 14028s pFCcGi or *E. coli* B/r pFCcGi in *D. discoideum* AX2 cnxA-GFP. Images of infected cells were acquired at different time points (1, 5, 15, and 23 h post infection) using a Zeiss LSM 710 laser scanning confocal microscope. The complete set of images captured during the experiment is shown in Supplementary Figure S1.

Red fluorescent bacteria were detected within amoeba after 1 h of co-incubation, indicating that both bacteria were actively internalized by *D. discoideum* (**Figure 2**; Supplementary Figure S1). In the case of *E. coli* B/r, intracellular bacteria were observed up to 5 h of co-incubation. After that time point, no bacteria were detected in association with amoeba (**Figure 2**; Supplementary Figure S1). In contrast, intracellular *S.* Typhimurium 14028s cells were observed at every time point evaluated. In fact, we were able to detect bacteria in substantial numbers within *D. discoideum* even after 23 h of co-incubation (**Figure 2**; Supplementary Figure S1). These observations confirmed that *S.* Typhimurium is able to survive and replicate intracellularly in *D. discoideum*, and allowed us to identify genes required for this process.

### An *aroA* Mutant of *S.* Typhimurium is Unable to Survive in *D. discoideum*

We developed a competition assay to evaluate the contribution of selected genes in the ability of *S.* Typhimurium to survive intracellularly in *D. discoideum*. In this assay, the internalization and intracellular survival of a defined mutant and the wild-type strain were directly compared when competing for *D. discoideum* as a replicative intracellular niche at different times of infection.

**FIGURE 3:**
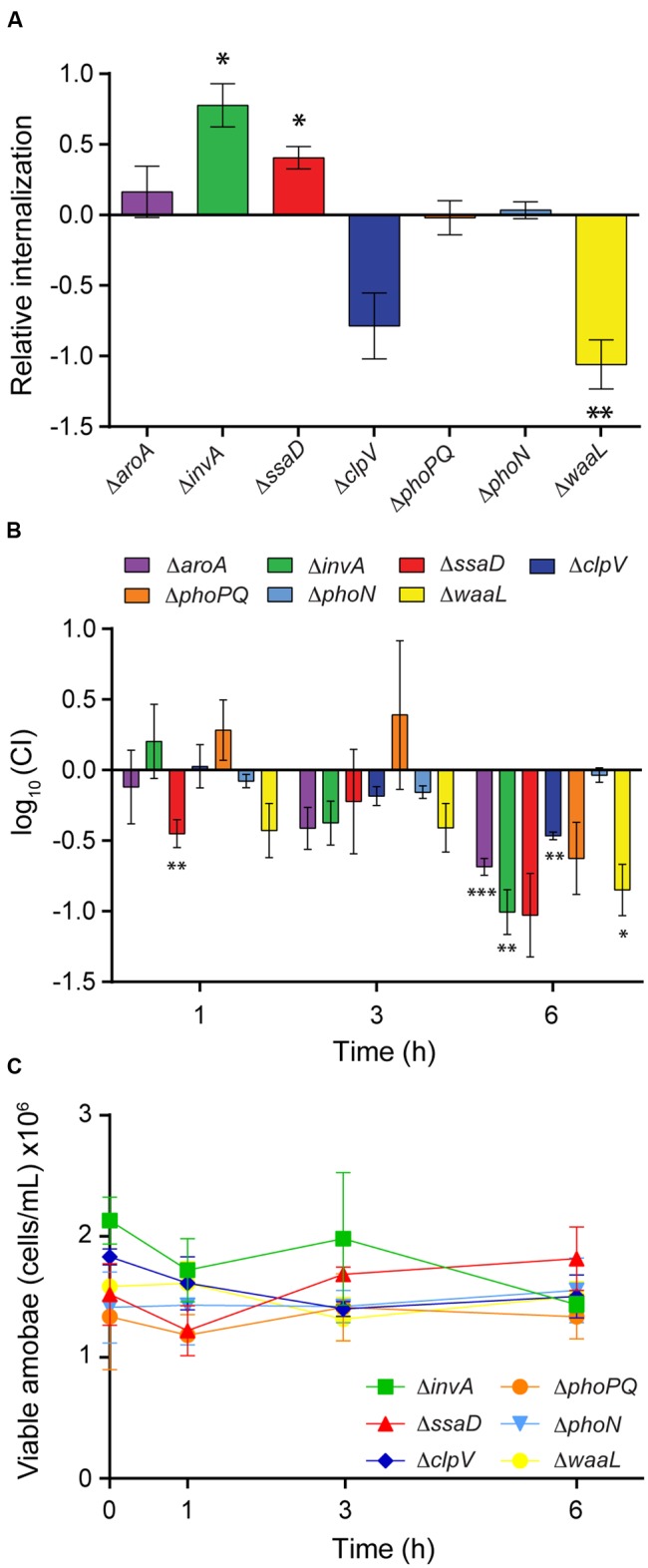
**Internalization and intracellular survival of *S.* Typhimurium 14028s and defined mutants in *D. discoideum.*** Competition assays were used to determine the intracellular survival of *S.* Typhimurium mutants Δ*aroA* (purple), Δ*invA* (green), Δ*ssaD* (red) Δ*clpV* (blue), Δ*phoPQ* (orange), Δ*phoN* (light blue), and Δ*waaL* (yellow). **(A)** Internalization relative to the wild-type strain calculated as (CFU_mutant_/CFU_WT_)_t=0_/(CFU_mutant_/CFU_WT_)_inoculum_ and expressed as log_10_. **(B)** Competitive index (CI) between a defined mutant and the wild-type strain calculated at each time point as (CFU_mutant_/CFU_WT_)_t__=__x_/(CFU_mutant_/CFU_WT_)_t=0_ and expressed as log_10_. **(C)** Population of viable amoebae at each time point expressed as cells/mL. Graphics shows the mean of at least three independent assays ± SEM. Statistical significance was determined using a two-tailed Student’s *t*-test (^∗^*P* < 0.05, ^∗∗^*P* < 0.005, ^∗∗∗^*P* < 0.001).

*Salmonella aroA* mutants are highly attenuated *in vivo* (Hoiseth and Stocker, 1981; Stocker et al., 1983; Cooper et al., 1990) and present strong defects in intracellular survival in macrophages *in vitro* (Fields et al., 1986; Lowe et al., 1999). Therefore, we aimed to determine if a Δ*aroA* mutant presents the same survival defect in *D. discoideum*. Using our competition assay, we observed that the Δ*aroA* mutant was internalized at wild-type levels (**Figure 3A**). However, the mutant strain was defective in intracellular survival in the amoeba at 6 h post infection (**Figure 3B**). No effect in amoeba viability was observed during the course of the experiment (**Figure 3C**).

### Relevant Genes Linked to *Salmonella* Virulence Are Required to Survive Intracellularly in *D. discoideum*

Considering that the intracellular survival defect shown by the Δ*aroA* mutant is associated to metabolic and envelope integrity defects (Sebkova et al., 2008), we included in our study a group of mutants in genes directly linked to *Salmonella* virulence in different models (i.e., Δ*invA*, Δ*ssaD*, Δ*clpV*, and Δ*phoPQ*). We chose genes *invA* and *ssaD* (also named *spiC*) because they encode essential structural components of T3SS_SPI-1_ and T3SS_SPI-2_, respectively (for a review see Moest and Meresse, 2013). In addition, *clpV* (also named *sciG*) encodes a chaperone essential for protein secretion through a type VI secretion system (T6SS; Bonemann et al., 2009; Pietrosiuk et al., 2011; Kapitein et al., 2013). We also chose genes *phoP* and *phoQ* because they encode a two-component system that regulates the expression of many virulence genes in *Salmonella* (Miller et al., 1989). As a control, we included mutants Δ*phoN* and Δ*waaL* in our experiments. *phoN* encodes an acid phosphatase (Kier et al., 1979; Kasahara et al., 1991) and null-mutants of this gene in *Salmonella* present wild-type levels of intracellular survival in macrophages (Klumpp and Fuchs, 2007) and systemic colonization in mice during competitive infections (Weening et al., 2005). Finally, *waaL* encodes the O-antigen ligase that is required for the production of a complete LPS structure (MacLachlan et al., 1991). It has been reported that *Salmonella waaL* mutants present defects in intracellular survival and are attenuated *in vivo* (Lyman et al., 1976; Aballay et al., 2003; Nagy et al., 2006; Duerr et al., 2009; Kong et al., 2011; Bender et al., 2013).

First, we evaluated the internalization of each mutant strain relative to the wild type during competitive infection. We observed that mutants Δ*invA* and Δ*ssaD* were internalized at higher levels than the wild-type strain (**Figure 3A**). These results suggest that *S.* Typhimurium can evade phagocytosis by *D. discoideum* through a mechanism that depends on the function of T3SS_SPI-1_ and T3SS_SPI-2_. On the other hand, mutants Δ*phoPQ*, Δ*clpV*, and Δ*phoN* were internalized at wild-type levels, while mutant Δ*waaL* was poorly internalized in comparison to the wild type (**Figure 3A**). Next, we evaluated the intracellular survival of each mutant strain relative to the wild type at different times post infection. As in the case of the Δ*aroA* strain, mutants Δ*invA*, Δ*ssaD*, Δ*phoPQ*, Δ*clpV*, and Δ*waaL* showed an impaired ability to survive intracellularly in *D. discoideum* (**Figure 3B**). In contrast, the intracellular survival of mutant Δ*phoN* in this organism was comparable to that shown by the wild-type strain (**Figure 3B**). No effect in amoeba viability was observed during the course of these experiments (**Figure 3C**).

## Discussion

In this study, we have confirmed that wild-type *S.* Typhimurium can survive intracellularly in the social amoeba *D. discoideum*. We adapted infection assays that allowed us to follow the bacterial population associated with amoebae by direct CFUs counts and by laser scanning confocal microscopy. Using both methodologies, we observed that *S.* Typhimurium 14028s survives at least for 24 h in *D. discoideum* under our experimental conditions. In contrast, *E. coli* B/r was unable to survive within this amoeba. It is worth mentioning that this bacterial strain is routinely used as food source for *D. discoideum* (Fey et al., 2007).

Most probably, the discrepancies between our results and early reports indicating that *Salmonella* is unable to survive in *D. discoideum* (Skriwan et al., 2002; Jia et al., 2009) are due to differences in the serovar of *Salmonella* characterized, the strain employed, and the use of infection assays based on the standard gentamicin-protection assay. Also, medium composition and general growth conditions have been implicated in the modulation of *Salmonella* virulence on *D. discoideum* (Sillo et al., 2011). In this study, we used a well-known virulent strain of *S.* Typhimurium, and long-term incubations with gentamicin were not used in our assays. This because during the setup of our infection protocols we realized that gentamicin readily enters in *Dictyostelium* cells, killing all intracellular bacteria after 6 h of infection (Supplementary Figure S2A). An attempt to use sodium azide instead of gentamicin to eliminate extracellular bacteria in our assays resulted in killing of amoeba, regardless of the presence of either *S.* Typhimurium 14028s or *E. coli* B/r during the assays (Supplementary Figure S2B). Finally, we decided to use gentamicin (10 mg/L) only in our competition assays during the first washing step before preparing the samples for intracellular CFU determination. The amount of gentamicin used corresponds to the minimum inhibitory concentration we determined for *S.* Typhimurium 14028s.

Other difference between our experimental conditions and those used by others is that we always used *D. discoideum* cells from axenic cultures in early exponential growth phase (cell densities of 1–2 × 10^6^ cells/mL, at most). The general recommendation is to use cells from axenic cultures in exponential phase (cell densities up to 4 × 10^6^ cells/mL; Fey et al., 2007). In early exponential phase cultures, we observed a greater proportion of single cells in comparison to cultures grown to higher densities, where cells tend to aggregate making more difficult to determine the exact amoeba population during our infections, even at early time points.

Using our competition assay, we observed that mutants Δ*aroA*, Δ*waaL*, Δ*invA*, Δ*ssaD*, Δ*clpV*, and Δ*phoPQ* present important defects in intracellular survival in *D. discoideum* when compared to the wild-type strain. Of note, the intracellular survival of a Δ*phoN* mutant was similar to the wild-type strain under the same experimental conditions. This result indicates that acid phosphatase PhoN is dispensable for survival of *S.* Typhimurium and *D. discoideum*, as reported in other infection models (Weening et al., 2005; Klumpp and Fuchs, 2007). In addition, having a mutant with no phenotype in our intracellular survival assay validates the observations made for mutants Δ*aroA*, Δ*waaL*, Δ*invA*, Δ*ssaD*, Δ*clpV*, and Δ*phoPQ*.

It is well documented that *aroA* mutants of *S.* Typhimurium and other *Salmonella* serovars are strongly attenuated in different models of infection (Hoiseth and Stocker, 1981; Stocker et al., 1983; Fields et al., 1986; Cooper et al., 1990; Lowe et al., 1999). In fact, we reported that a Δ*aroA* mutant of *S.* Enteritidis presents strong defects in systemic colonization of BALB/c mice and intracellular survival in RAW264.7 murine macrophages *in vitro* (Silva et al., 2012). Most probably, these phenotypes and the intracellular survival defect observed in *D. discoideum* are directly linked to deficient biosynthesis of aromatic compounds and defects in the integrity of the cell wall and the outer membrane presented by *aroA* null-mutants of *Salmonella* (Sebkova et al., 2008).

Although there are studies on the role played by O-antigen in the prey discrimination for serovars of *Salmonella* presented by a number of intestinal amoebae from different hosts (Wildschutte et al., 2004; Wildschutte and Lawrence, 2007), this is the first report on the contribution of O-antigen to the intracellular survival of *Salmonella* in amoebae. Despite this, it has been reported that *Salmonella waaL* mutants present defects in intracellular survival in murine enterocytes (Duerr et al., 2009) and are attenuated *in vivo* in different models of infection, including mice (Lyman et al., 1976; Kong et al., 2011), *Caenorhabditis elegans* (Aballay et al., 2003; Tenor et al., 2004) and *Galleria mellonella* (Bender et al., 2013). Most probably, the intracellular defect presented by our Δ*waaL* mutant in *D. discoideum* is linked to defects in motility and susceptibility to antimicrobial substances, such as cationic peptides (Nagy et al., 2006; Kong et al., 2011).

T3SS_SPI-1_ and T3SS_SPI-2_ inject effector proteins into eukaryotic cells (including epithelial and phagocytic cells) and are critical during *Salmonella* infection of animal hosts (reviewed in Haraga et al. (2008). In addition, PhoP/PhoQ two-component system regulates the expression of many virulence genes following *Salmonella* uptake into phagosomes (Groisman et al., 1989; Miller et al., 1989; Alpuche Aranda et al., 1992), including genes in SPI-2 (Bijlsma and Groisman, 2005). Furthermore, T3SS_SPI-2_ and PhoP/PhoQ are critical for intracellular survival of *Salmonella* serovars in macrophages (Groisman et al., 1989; Miller et al., 1989; Cirillo et al., 1998; Hensel et al., 1998). Regarding their role during *Salmonella*-protozoa interaction, it has been reported that T3SS_SPI-2_ and PhoP/PhoQ are required for survival of *S.* Typhimurium strain F98 in *A. polyphaga*, while T3SS_SPI-1_ appears to be dispensable for this process (Bleasdale et al., 2009). Another study also indicates that T3SS_SPI-1_ is not required for entry and survival of *S.* Dublin in *A. rhysodes* (Tezcan-Merdol et al., 2004).

In *S.* Typhimurium, *clpV* is located in SPI-6 as part of a gene cluster encoding a T6SS (Blondel et al., 2009; Mulder et al., 2012), and ClpV is essential for the secretion of proteins through the T6SS apparatus (Bonemann et al., 2009; Pietrosiuk et al., 2011; Kapitein et al., 2013). *Salmonella* mutants harboring deletions of *clpV* or T6SS loci present defects in intracellular survival in macrophages (Haneda et al., 2009; Mulder et al., 2012; Blondel et al., 2013) and systemic colonization in mice and chicken (Mulder et al., 2012; Pezoa et al., 2013, 2014). It has been reported that bacterial pathogens like *Vibrio cholerae* and *Burkholderia cenocepacia* require specific T6SS to survive predation by *D. discoideum* (Pukatzki et al., 2006; Aubert et al., 2008; Zheng et al., 2011). However, to the best of our knowledge this is the first report on the requirement of a T6SS in the intracellular survival of *Salmonella* in amoebae.

In addition to the mentioned phenotypes, we observed that mutants Δ*invA* and Δ*ssaD* were internalized in higher numbers than the wild type during competitive infections. These results suggest that T3SS_SPI-1_ and T3SS_SPI-2_ are required by *S.* Typhimurium to evade phagocytosis by *D. discoideum*. A similar observation was reported when the internalization of a Δ*invC* mutant of *S.* Typhimurium was assessed in dendritic cells (Bueno et al., 2010). As in the case of *invA, invC* encodes an essential component of T3SS_SPI-1_. The authors reported that, although being impaired for invasion of non-phagocytic cells (i.e., L-cells and MLE-12 cells), the Δ*invC* mutant was internalized at higher rates than the wild type by murine dendritic cells (Bueno et al., 2010). In addition, the Δ*invC* mutant was as proficient as the wild-type strain in blocking antigen presentation by infected dendritic cell to T cells *in vitro*. Thus, these observations suggest that *S.* Typhimurium employs T3SS_SPI-1_ to avoid phagocytosis by professional phagocytic cells such as *D. discoideum* and dendritic cells. Further studies are required to elucidate the role played by T3SS_SPI-2_ in this process, and to identify the effectors involved.

Furthermore, the plant growth-promoting rhizobacterium *Pseudomonas fluorescens* F113 harbors two independent T3SS, one of which is phylogenetically related to *Salmonella* T3SS_SPI-1_. It has been reported that mutants in genes *spaS* (encoding an essential component of the secretion apparatus) and *gacA* (encoding a positive regulator of *hilA* expression that is orthologous to SirA in *Salmonella*) are ingested preferentially by *A. polyphaga* when they are co-incubated with the wild-type strain (Barret et al., 2013). These results in *P. fluorescens* F113 and our results in *S.* Typhimurium suggest a common role for T3SS_SPI-1_ in resistance to amoeboid grazing. This phenomenon is similar to the preferential feeding of *A. castellanii* on non-toxic *gacS* mutants of *P. protegens* CHA0 (Jousset et al., 2009, 2010), suggesting that effectors secreted by different bacteria through T3SS related to *Salmonella* T3SS_SPI-1_ are toxic for amoebae. Further studies are required to confirm this hypothesis.

Altogether, we identified genes required for the intracellular survival of *S.* Typhimurium in *D. discoideum* that are associated with virulence in other infection models. These genes include those directly liked to functional biosynthesis of aromatic compounds, ligation of O-antigen to the LPS structure, secretion through T3SS_SPI-1_, T3SS_SPI-2_, and T6SS_SPI-6_, and PhoP/PhoQ two-component system. Therefore, our results indicate that *Salmonella* exploits a common set of genes and molecular mechanisms to survive within phagocytic amoeba and host cells, such us macrophages. It is worth mentioning that the genes included in this study represent only a small fraction of those involved in the internalization and intracellular survival of *Salmonella* in amoebae. Recently, in collaboration with other groups we generated different collections of defined single-gene and multi-gene deletion mutants in the genome of *S.* Typhimurium 14028s (Santiviago et al., 2009; Porwollik et al., 2014). High-throughput analyses of pools of mutants in some of these collections have been conducted in different models of infection, including regular BALB/c mice (Santiviago et al., 2009; Silva-Valenzuela et al., 2016b), tumor-bearing BALB/c mice (Silva-Valenzuela et al., 2016a), calves (Elfenbein et al., 2013), and chicks (Yang et al., 2016). Thus, the competition assay developed in this work will allow us to analyze our collections of mutants in order to define the gene complement required for *S.* Typhimurium to survive within *D. discoideum*. Experiments in this direction are currently on their way in our laboratory.

## Author Contributions

Conceived and designed the experiments: CS, FC, SR, MV, and CV. Performed the experiments: SR, MV, CV, PV, NC, PA, AS, and BL. Analyzed the data: CS, FC, SR, MV, CV, PV, NC, PA, AS, BL, and SA. Contributed reagents/materials/analysis tools: CS, FC, and SA. Wrote the paper: CS, SR, MV, and CV. All authors read and approved the final manuscript.

## Conflict of Interest Statement

The authors declare that the research was conducted in the absence of any commercial or financial relationships that could be construed as a potential conflict of interest.

## Footnotes

1 http://dictybase.org
