# Supplementary material for: Relevant Genes Linked to Virulence Are Required for Salmonella Typhimurium to Survive Intracellularly in the Social Amoeba Dictyostelium discoideum
Source: Front Microbiol. 2016 Aug 23;7:1305. doi: 10.3389/fmicb.2016.01305 (PMC4993766; doi:10.3389/fmicb.2016.01305)
Supplement: Supplementary file 1 [file Table_1.PDF]

Table S1.- Primers used in this study.

**Name**	**Sequence**
aroA_H1+P1	GTTGAGTTTCATGGAATCCCTGACGTTACAACCCATCGCGGTGCAGGCTGGAGCTGCTTC
aroA_H2+P2	AACAGAAGACTTAGGCAGGCGTACTCATTCGCGCCAGTTGCATATGAATATCCTCCTTAG
aroA_Out5	GCGCGCCTCTATCTATAACG
invA_H1+P1	GATACCTATAGTGCTGCTTTCTCTACTTAACAGTGCTCGTGTGCAGGCTGGAGCTGCTTC
invA_H2+P2	AATTAAGCCCTTATATTGTTTTTATAACATTCACTGACTTCATATGAATATCCTCCTTAG
invA_Out5	TGAGGGTTCGCTATTAACCG
ssaD_H1+P1	GTAGTAAATAATGGCATATCTCATGGTTAATCCAAAGAGTGTGCAGGCTGGAGCTGCTTC
ssaD_H2+P2	CATTTTCCACTCACTTAAAATCTAATGGATAGTTAATCAACATATGAATATCCTCCTTAG
ssaD_Out5	CGGTGGTGCTAGTGGTTTTT
phoQ_H1+P1	GGGAGAAGAGATGATGCGCGTACTGGTTGTAGAGGATAATGTGCAGGCTGGAGCTGCTTC
phoP_H2+P2	ACAGAAATGTTTATTCCTCTTTCTGTGTGGGATGCTGTCGCATATGAATATCCTCCTTAG
phoQ_Out5	CATCTGCTTGACGAACTGGA
phoN_H1+P1	GTGAGTCTTTATGAAAAGTCGTTATTTAGTATTTTTTCTAGTGTAGGCTGGAGCTGCTTC
phoN_H2+P2	ACTTTCACCTTCAGTAATTAAGTTCGGGGTGATCTTCTTTCATATGAATATCCTCCTTAG
phoN_Out5	GTCCGGTATGGACAGACGAT
clpV_H1+P1	GGCATAACACATGGAAACTCCTGTTTCACGCAGTGCGTTGGTGTAGGCTGGAGCTGCTTC
clpV_H2+P2	ACGGCCGGTTTCAGCAAACGATCTCAAAAACAATCTGCTCCATATGAATATCCTCCTTAG
clpV_Out5	GGCGGCAGTAAATACGATGT
WwaaL1	TCTATTTCTTAGCGCCAGCAGAAAACCGGTAATGATACCACATATGAATATCCTCCTTAG
WwaaL2	CTAACCACATCATTAACGTTAAATAAAGAGAAATGGAAGGTGTAGGCTGGAGCTGCTTCG
waaL-2	GTATCGGTTGATACCGGCCT
K1	CAGTCATAGCCGAATAGCCT
pCLF4_P1_Bam	CGGGATCCGGACTGGCTTTCTACGTGTTCC

Underlined sequences correspond to the region that anneals to the 5' or 3' end of the antibiotic-resistance cassette in template vectors pCLF1, pCLF2 and pCLF4.
