# Supplementary material for: Relevant Genes Linked to Virulence Are Required for Salmonella Typhimurium to Survive Intracellularly in the Social Amoeba Dictyostelium discoideum
Source: Front Microbiol. 2016 Aug 23;7:1305. doi: 10.3389/fmicb.2016.01305 (PMC4993766; doi:10.3389/fmicb.2016.01305)
Supplement: Supplementary file 2 [file Image_1.PDF]

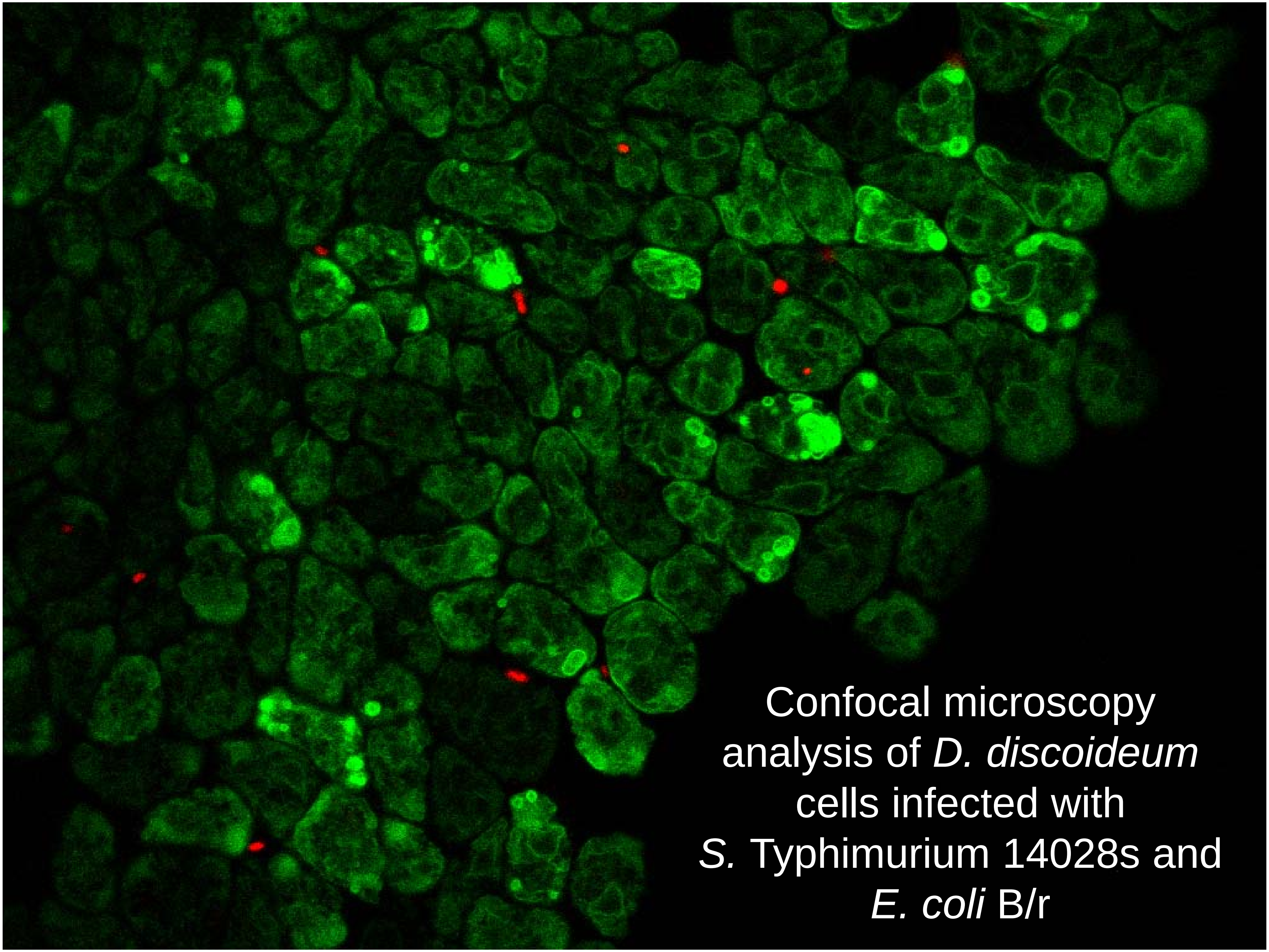

Confocal microscopy analysis of *D. discoideum* cells infected with *S.* Typhimurium 14028s and *E. coli* B/r

Figure S1.- Confocal microscopy analysis of *D. discoideum* cells infected with *S.* Typhimurium 14028s and *E. coli* B/r.

S. Typhimurium 14028s and *E. coli* B/r were transformed by electroporation with plasmid pFCcGi encoding the red fluorescent protein mCherry expressed constitutively (Figueira et al., 2013). Axenic *D. discoideum* AX2 cnxA-GFP cells (~2 x 10^7 cells) were co-incubated with each bacteria at 22°C for 24 h in 10 mL of Soerensen buffer, using an MOI of 10 bacteria/amoeba. Images of infected cells were acquired every hour using a Zeiss LSM 710 laser scanning confocal microscope equipped with a 63x 1.4 NA optic setup. Prior to observation, cells were mounted on a thin layer of 1% agarose in PBS buffer deposited on a glass slide. To visualize GFP-associated fluorescence (amoebae), the sample was excited at 488 nm with an argon laser and emission was detected using a filter in the 493-549 nm range. To visualize mCherry-associated fluorescence (bacteria), the sample was excited at 543 nm with a HeNe laser and emission was detected using a filter in the 548-679 nm range. Images were acquired using the ZEN 2012 Black software (Zeiss), and analyzed using Fiji and ImageJ softwares (Schindelin et al., 2012; Schneider et al., 2012).

E. coli B/r

S. Typhimurium 14028

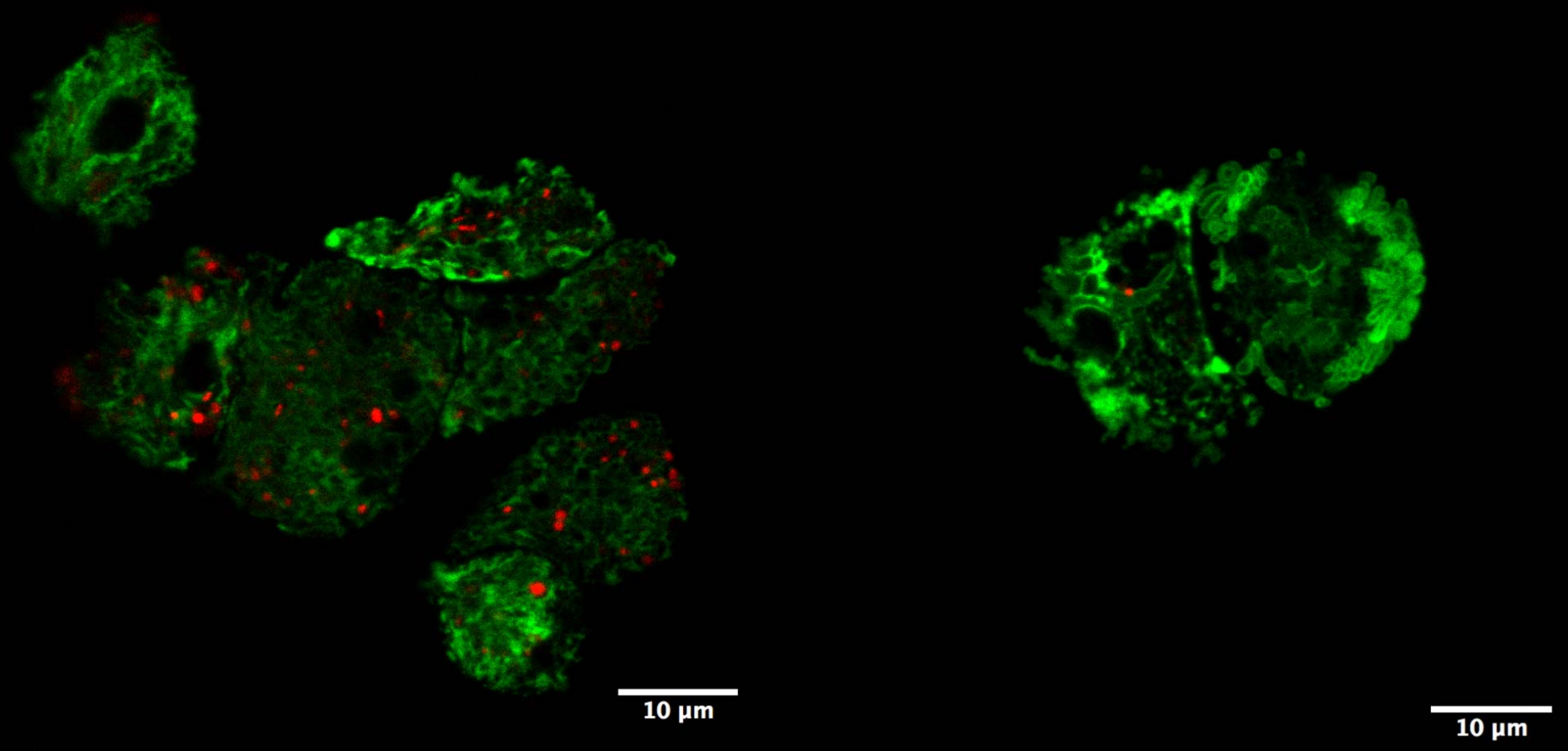

t = 1h

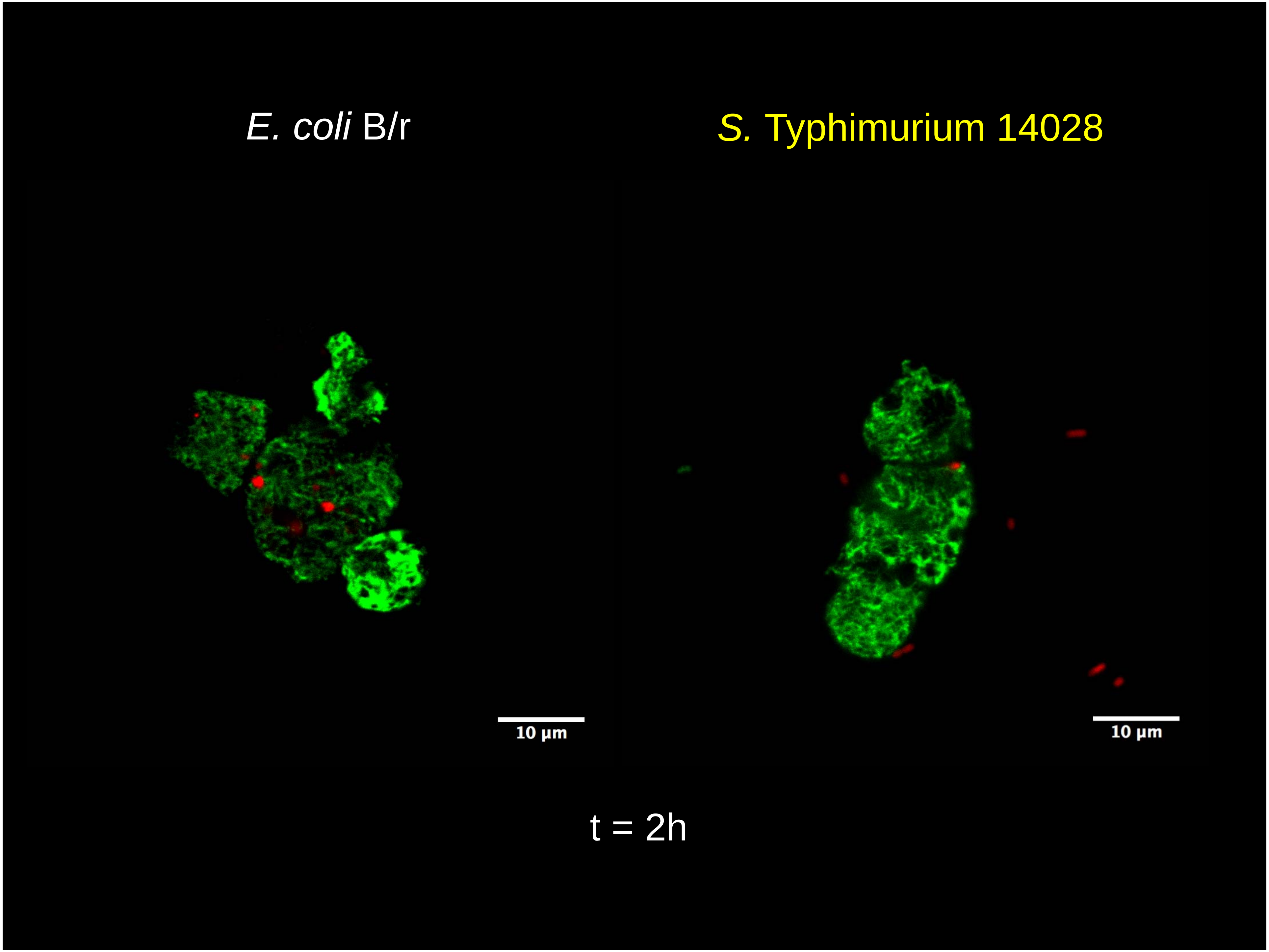
E. coli B/r
S. Typhimurium 14028
10 µm
10 µm
t = 2h

E. coli B/r

S. Typhimurium 14028

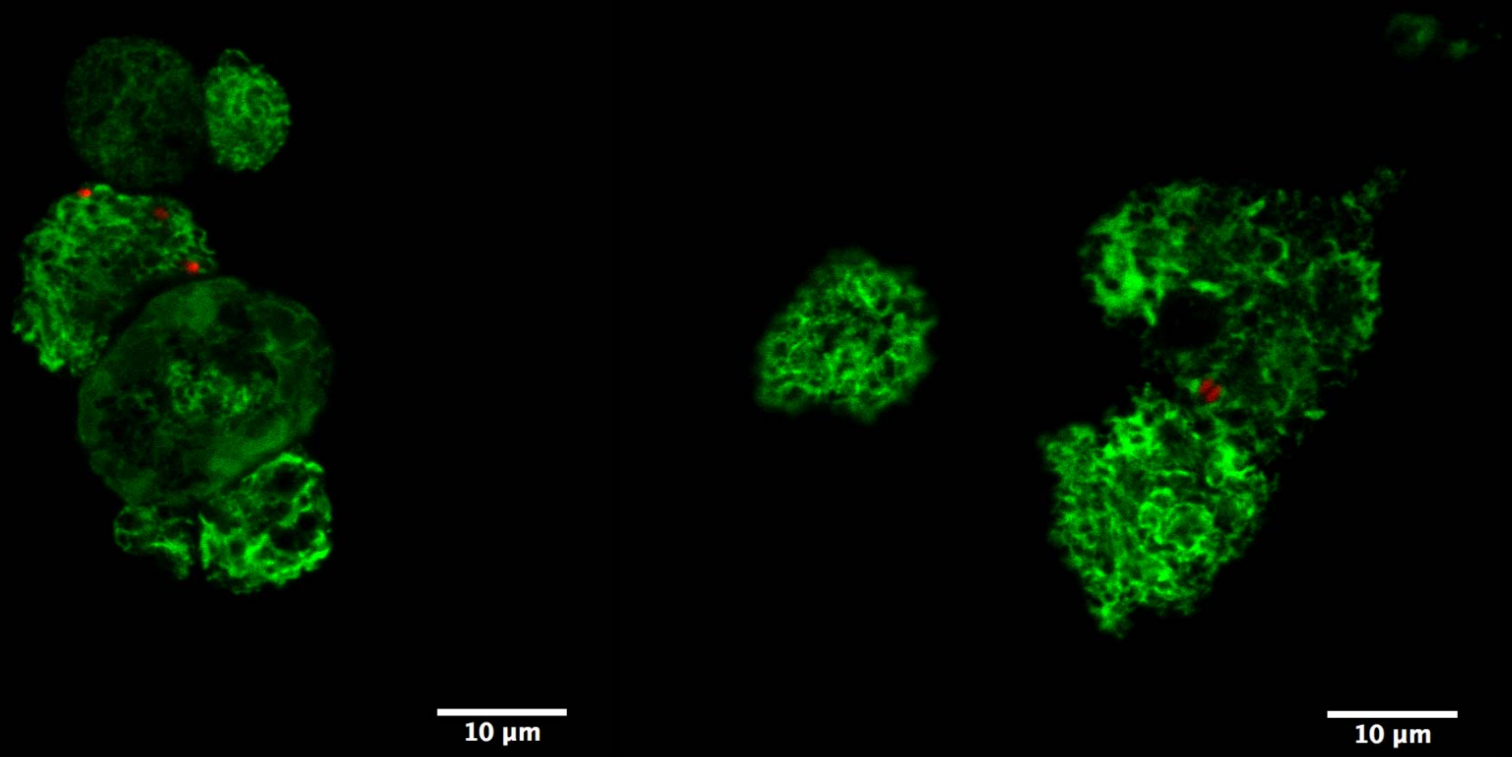

t = 3h

E. coli B/r

S. Typhimurium 14028

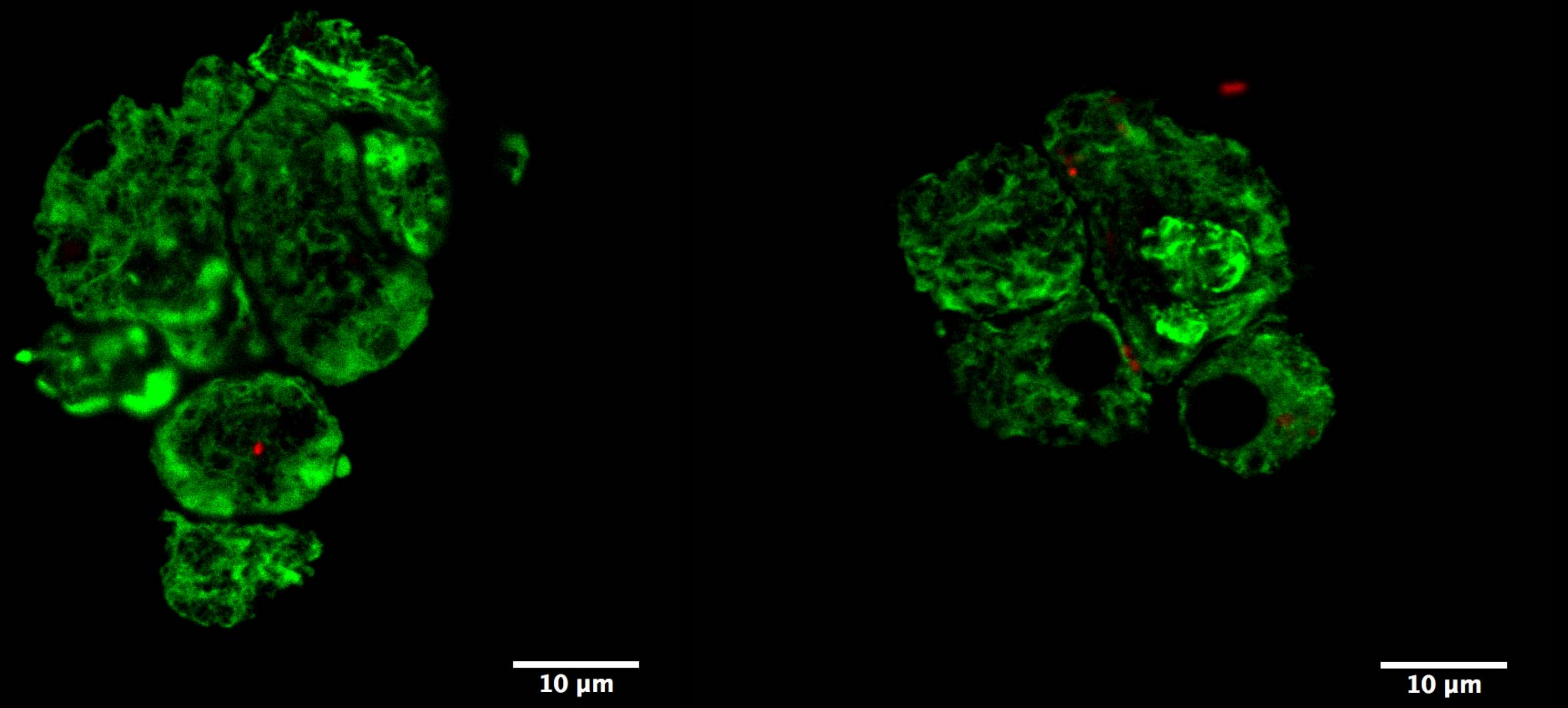

t = 4h

E. coli B/r *S.* Typhimurium 14028

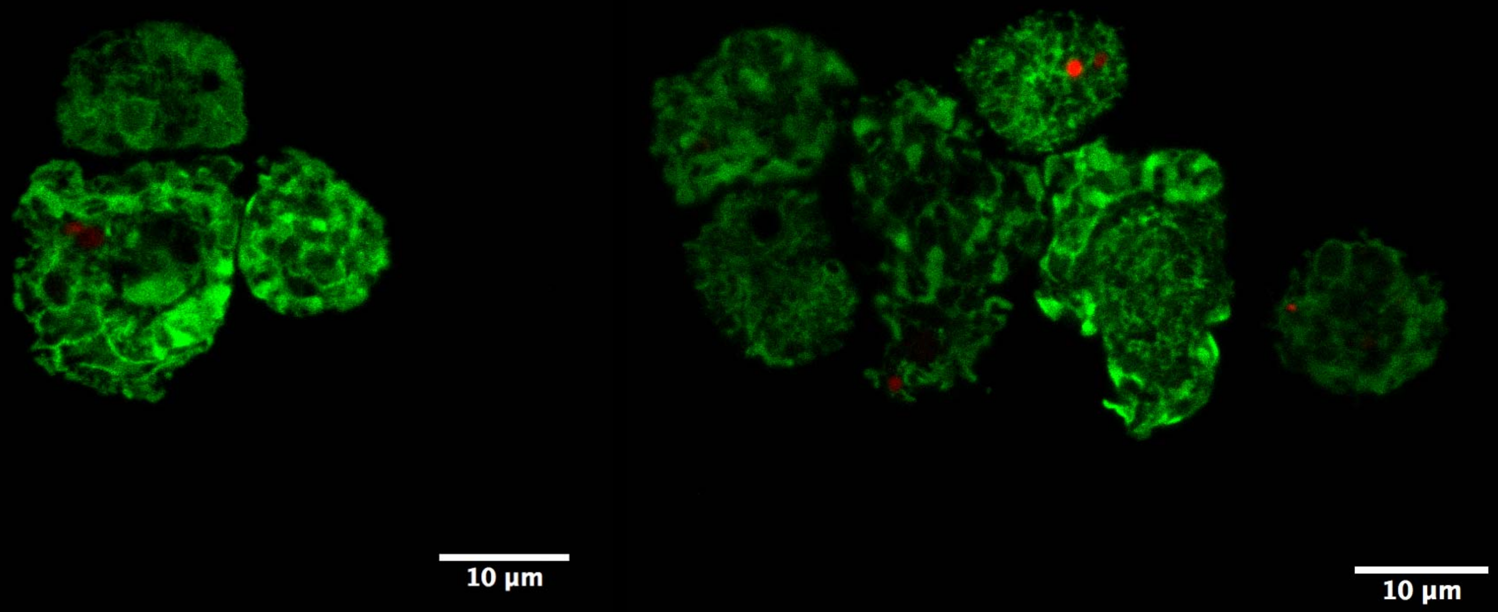

t = 5h

E. coli B/r

S. Typhimurium 14028

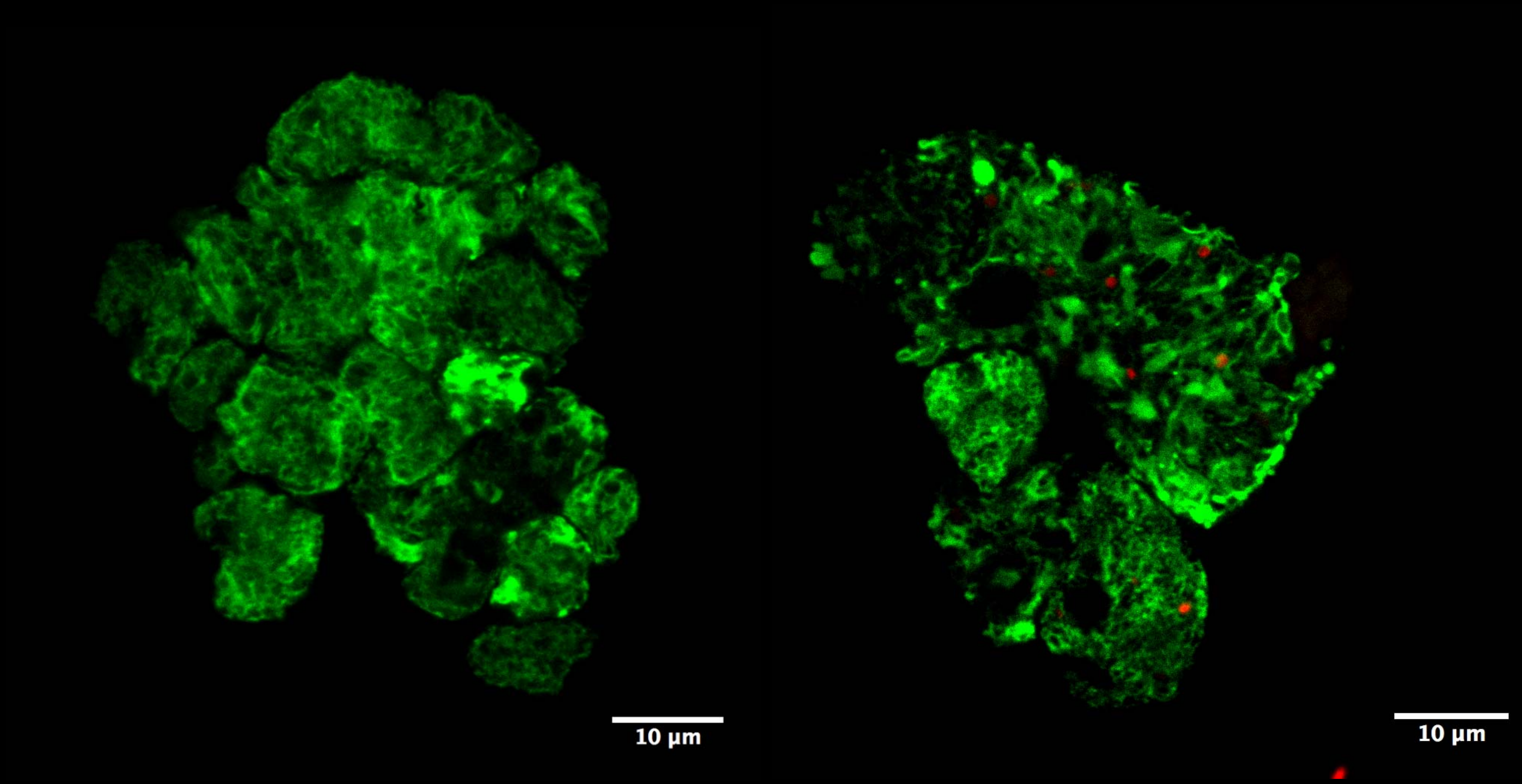

t = 6h

E. coli B/r

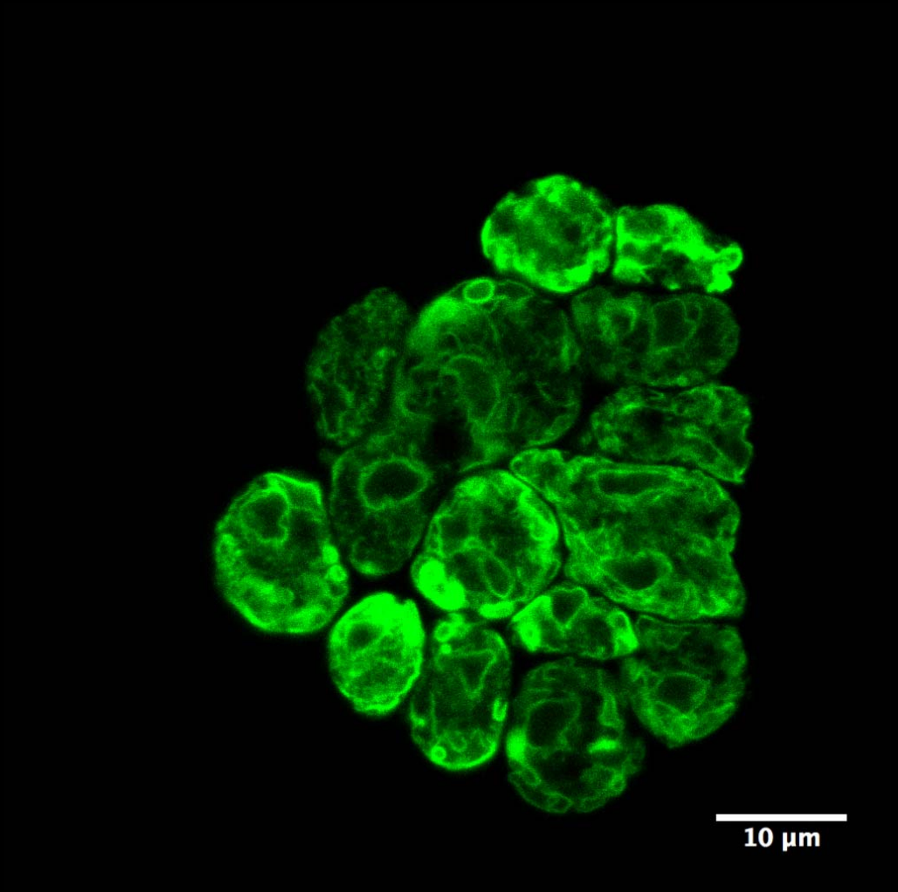

S. Typhimurium 14028

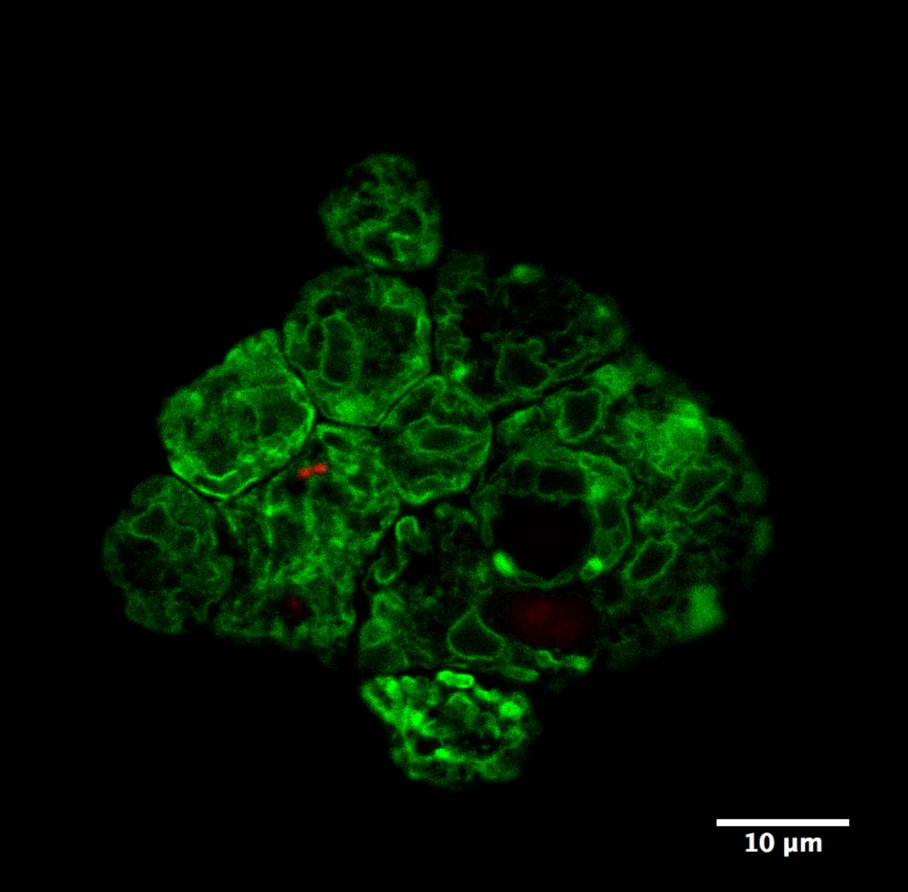

t = 8h

E. coli B/r

S. Typhimurium 14028

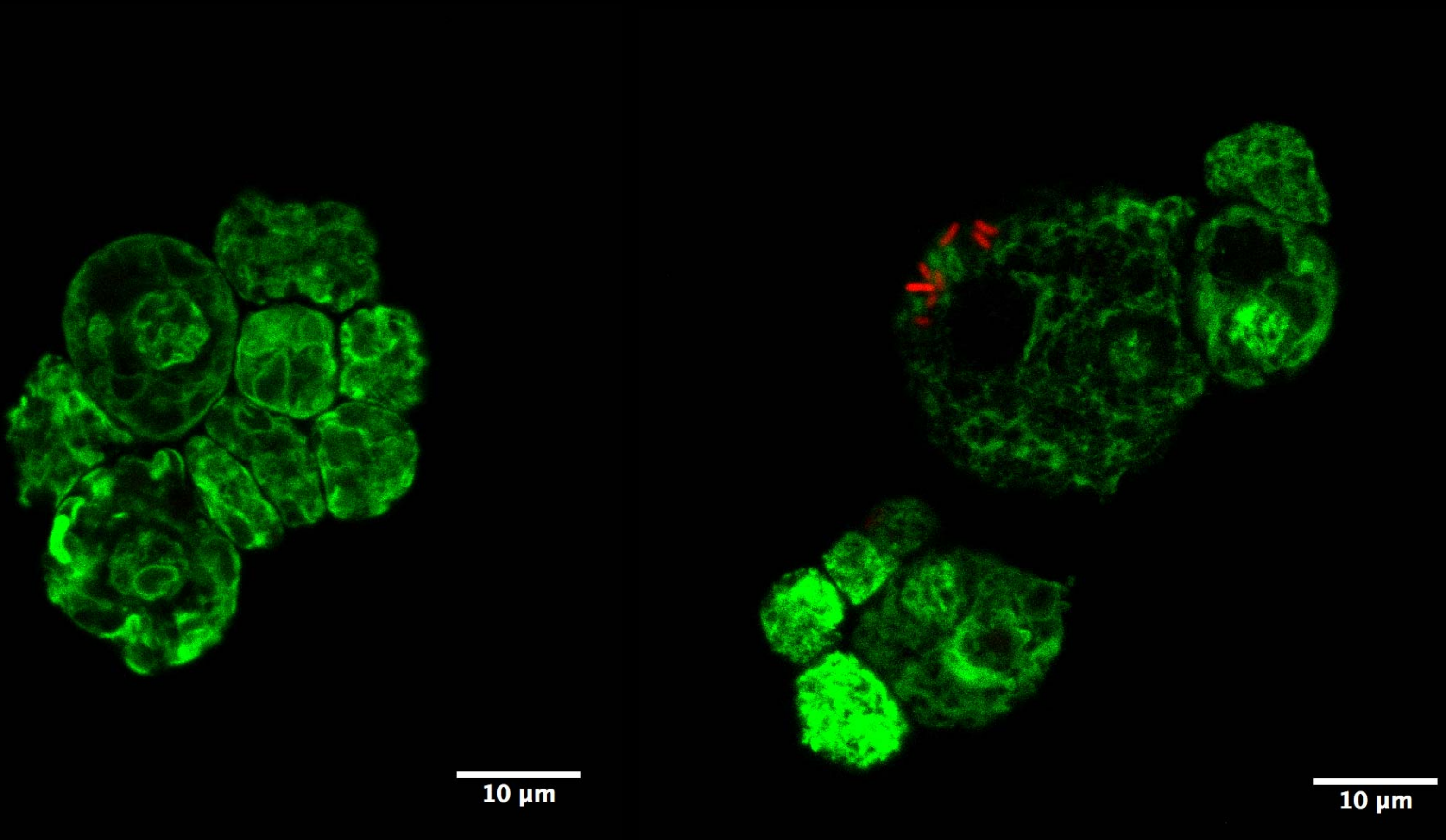

t = 11h

E. coli B/r

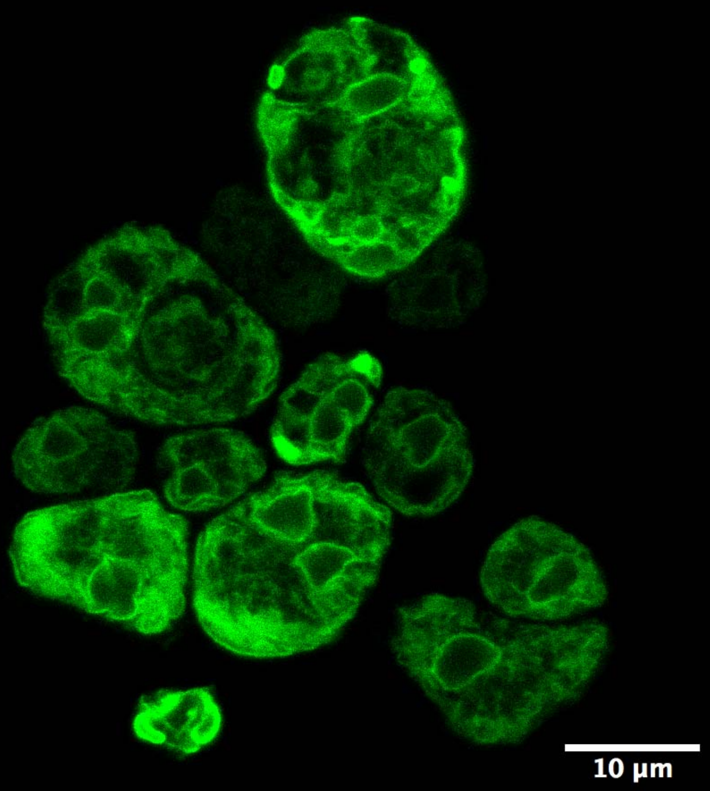

S. Typhimurium 14028

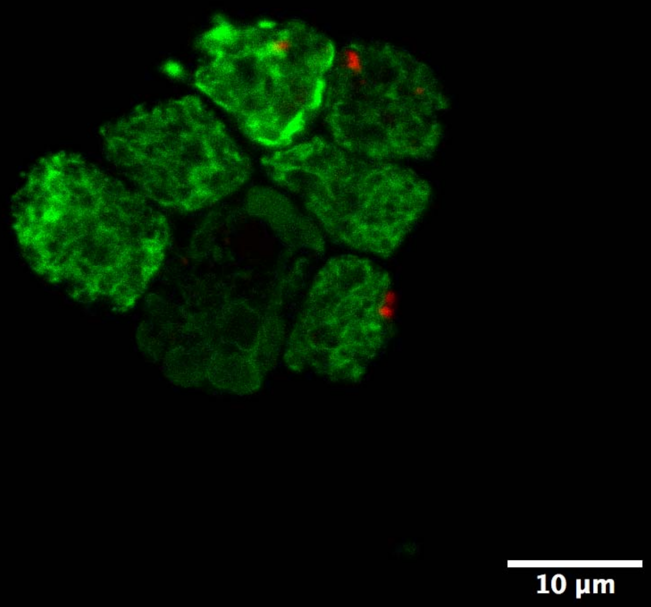

t = 13h

E. coli B/r

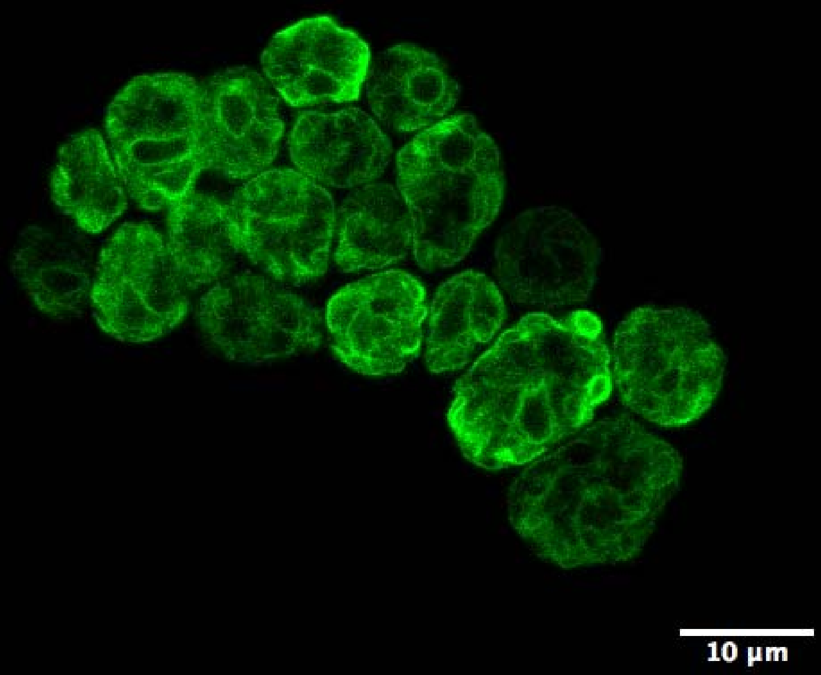

S. Typhimurium 14028

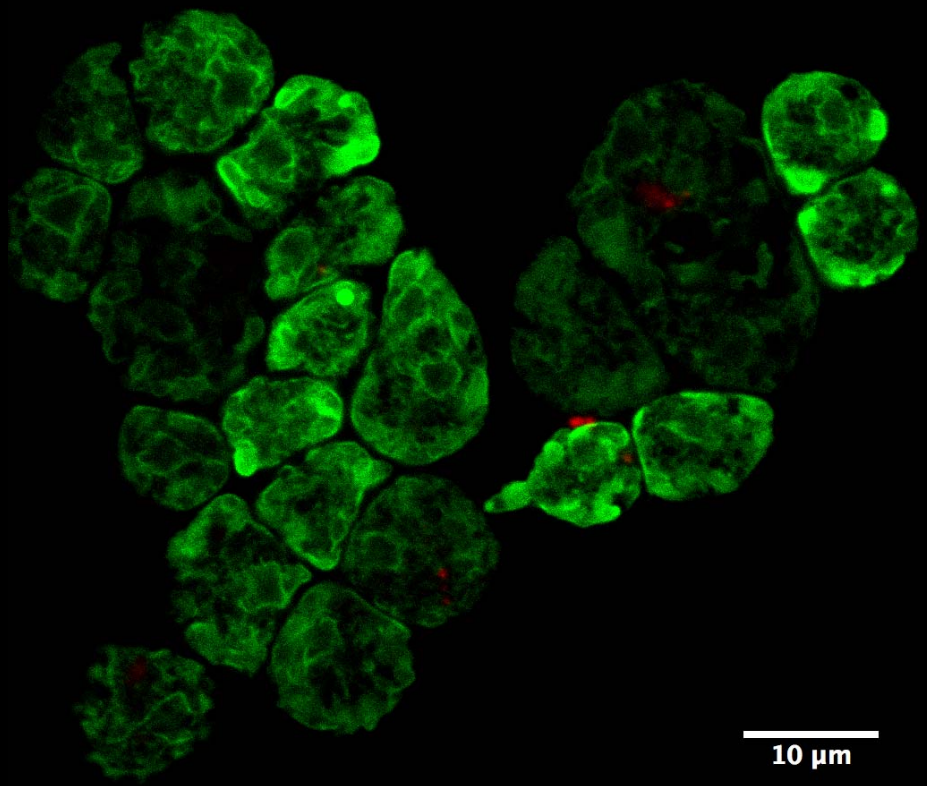

t = 15h

E. coli B/r

S. Typhimurium 14028

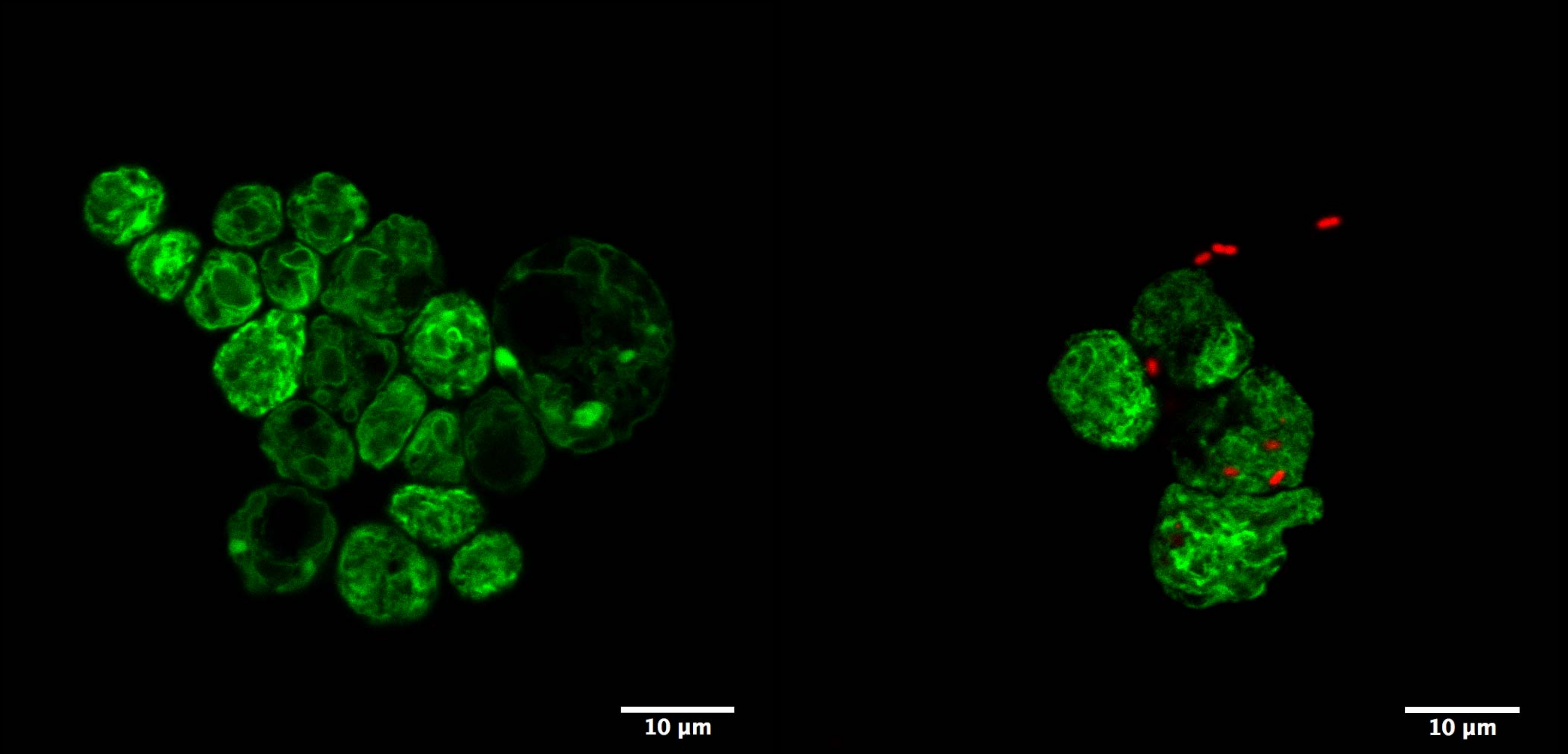

t = 17h

E. coli B/r

S. Typhimurium 14028

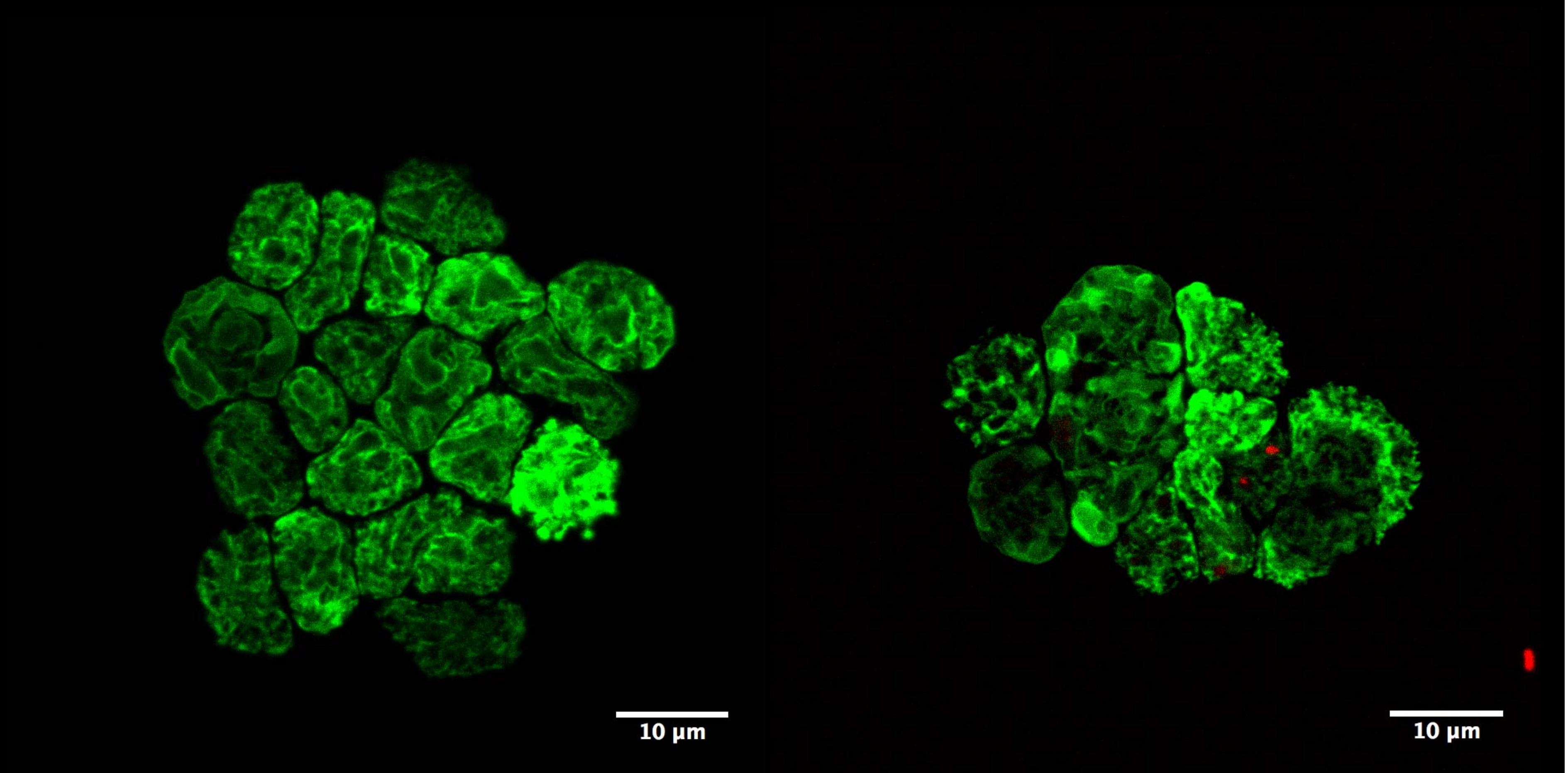

t = 23h
