# Supplementary material for: Relevant Genes Linked to Virulence Are Required for Salmonella Typhimurium to Survive Intracellularly in the Social Amoeba Dictyostelium discoideum
Source: Front Microbiol. 2016 Aug 23;7:1305. doi: 10.3389/fmicb.2016.01305 (PMC4993766; doi:10.3389/fmicb.2016.01305)
Supplement: Supplementary file 3 [file Image_2.PDF]

A

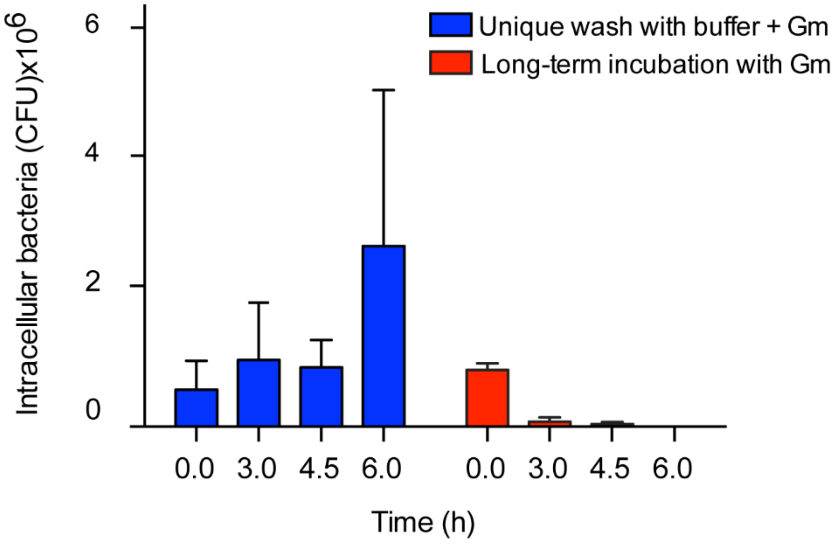

B

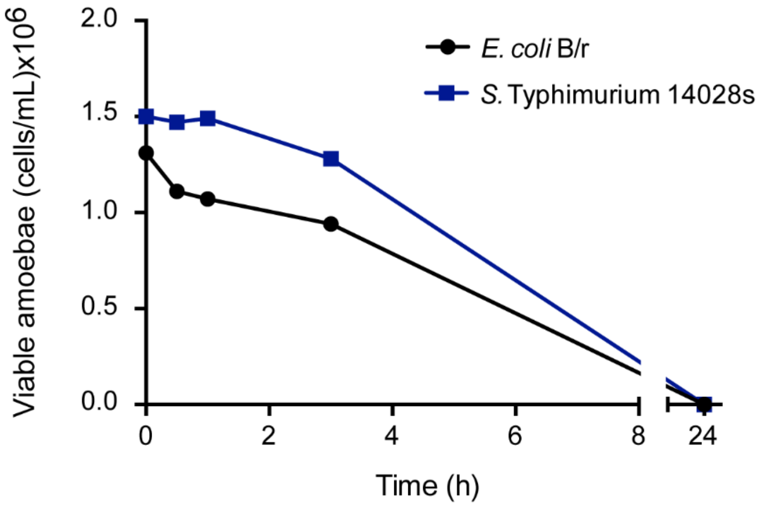

Figure S2.- Effect of gentamicin or sodium azide treatment on the viability of intracellular bacteria and *D. discoideum* during infection.

(A) Effect of gentamicin treatment on viability of intracellular bacteria. Axenic *D. discoideum* AX4 cells (~2 x 10^7 cells) were co-incubated with *S*. Typhimurium 14028s at 22°C with agitation (180 rpm) in 10 mL of Soerensen buffer using a MOI of 100 bacteria/amoeba. After 1 h of co-incubation, amoebae were washed three times in Soerensen buffer to remove extracellular bacteria. For the “Unique wash with buffer + Gm” group, infected cells were suspended in 10 mL of Soerensen buffer (t=0) and further incubated. Aliquots were obtained at 0, 3, 4.5 and 6 h post infection. At each time point, infected amoebae were washed in Soerensen buffer supplemented with gentamicin (10 mg/L), then washed in Soerensen buffer to remove the antibiotic, and finally lysed with 0.2% Triton X-100. For the “Long-term incubation with Gm” group, infected cells were suspended in 10 mL of Soerensen buffer containing gentamicin (20 mg/mL) (t=0) and further incubated at Soerensen buffer. Aliquots were obtained at 0, 3, 4.5 and 6 h post infection. Infected amoebae recovered at each time point, were washed in Soerensen buffer to remove the antibiotic, and lysed with 0.2% Triton X-100. Titers of intracellular bacteria were determined by serial dilutions and plating on LB agar supplemented with the appropriate antibiotics.

(B) Effect of sodium azide treatment on viability of *D. discoideum*. Axenic *D. discoideum* AX4 cells (~2 x 10^7 cells) were co-incubated with *S*. Typhimurium 14028s or *E. coli* B/r at 22°C with agitation (180 rpm) in 10 mL of Soerensen buffer using a MOI of 100 bacteria/amoeba. After 1 h of co-incubation, amoebae were washed with 0.2% sodium azide in Soerensen buffer to remove extracellular bacteria. Then, infected cells were suspended in 10 mL of Soerensen buffer (t=0) and further incubated at 22°C with agitation. Aliquots were obtained at 0, 0.5, 1, 3 and 24 h post infection and viable amoebae were determined at each time point by Trypan blue exclusion and counting on a Neubauer chamber.
